# Pregnancy Outcome After Vaginal Natural Orifice Transluminal Endoscopic Surgery: A Follow-Up Retrospective Observational Cohort Study

**DOI:** 10.7759/cureus.111706

**Published:** 2026-06-29

**Authors:** Karen Lens, Andrea Stuart, Jan Baekelandt

**Affiliations:** 1 Department of Gynecology, Imelda Hospital, Bonheiden, BEL; 2 Department of Obstetrics and Gynecology, Institute for Clinical Sciences, Lund University, Lund, SWE

**Keywords:** adnexectomy, cystectomy, myomectomy, pregnancy outcome, salpingectomy, salpingostomy, vnotes

## Abstract

Study objective

Vaginal natural orifice transluminal endoscopic surgery (vNOTES) is a minimally invasive surgical technique, with improved cosmetic results, reduced operation time, and reduced postoperative pain as its main advantages. The aim of the study was to assess pregnancy outcomes and the safety of vaginal delivery after fertility-sparing vNOTES procedures.

Methods

A retrospective, observational, single-centre cohort study was performed between January 2014 and March 2023. Fertility-sparing surgeries performed by vNOTES, including salpingostomy, salpingectomy, cystectomy, unilateral adnexectomy, and myomectomy for benign indications, were included. A total of 191 patients underwent vNOTES procedures, of whom 42 subsequently became pregnant, resulting in 52 pregnancies with deliveries between November 2015 and February 2024.

Results

The population consisted of an obstetrical high-risk population undergoing fertility-sparing surgery, mainly secondary to infertility or ectopic pregnancies. Among the 42 patients who became pregnant after a vNOTES procedure, 32 pregnancies occurred within the first year after vNOTES (76.2%). Assisted reproductive treatment was performed in 23.1% of all 52 pregnancies. Four patients delivered preterm, but the indication could not be linked to previous vNOTES procedures.

Vaginal delivery occurred in 35 (67.3%) cases, and caesarean section (CS) was performed in 17 (32.7%) cases. A high percentage (21.2%) of these CS were elective, and none were a result of previous vNOTES procedures. Trial of labour after caesarean (TOLAC) was observed in four (7.7%) pregnancies, resulting in two uncomplicated vaginal deliveries and two CS due to cephalopelvic disproportion or foetal distress. Among patients with vaginal delivery, no tearing of the posterior colpotomy scar was described, and no grade 3 or 4 perineal ruptures were observed.

Conclusions

This is the largest known cohort evaluating the safety of pregnancy and vaginal delivery after vNOTES in a high-risk population. No pregnancy-related complications were reported as a result of previous vNOTES surgery. The posterior colpotomy, necessary for performing vNOTES, appears to be safe for a full-term pregnancy and during vaginal delivery. Therefore, previous vNOTES is not an indication for an elective CS.

## Introduction

Vaginal natural orifice transluminal endoscopic surgery (vNOTES) is a minimally invasive surgical technique in which access to the abdominal cavity is obtained through a posterior colpotomy. In recent years, vNOTES has quickly gained ground due to its broad range of indications and multiple benefits compared to conventional laparoscopy. Improved cosmetic results, reduced operation time, and reduced postoperative pain are described as the main advantages, resulting in shorter hospitalisation time [1-3]. The safety and feasibility of vNOTES as a surgical technique have already been established [3-6], and it has also been shown to be safe in comparison with conventional laparoscopy [3,7]. However, only limited data are available regarding the safety of full-term pregnancy and vaginal delivery after vNOTES.

We conducted a follow-up study of an earlier retrospective observational cohort study by Tavano et al. [8], in which no negative impact on pregnancy or obstetrical outcome was observed. We used the same study design to test their hypothesis, though in a cohort twice as large. To the best of our knowledge, this is the largest cohort to date to assess this subject. We aimed to provide information about multiple pregnancy outcomes and insight into the safety of vaginal delivery after fertility-sparing vNOTES procedures. 

## Materials and methods

Study design 

We conducted a follow-up, retrospective, single-centre observational cohort study following the same study design as the initial cohort study by Tavano et al. [8]. The study was performed at Imelda Hospital in Bonheiden, Belgium, covering a period of almost 10 years, including vNOTES procedures performed between January 2014 and March 2023. Ethical approval was obtained from the local Institutional Ethics Committee.

Only fertility-sparing procedures performed by vNOTES were eligible for inclusion: unilateral adnexectomy, cystectomy, unilateral salpingectomy, salpingostomy, myomectomy, or procedures for diagnostic purposes. Other vNOTES procedures, including hysterectomy, bilateral adnexectomy, or bilateral salpingectomy, were excluded from analysis. In addition, only patients younger than 43 years at the time of vNOTES were considered. All procedures were performed by the same surgeon with extensive experience in vNOTES.

A total of 191 patients met the inclusion criteria, resulting in 52 pregnancies in a total of 42 patients with deliveries between November 2015 and February 2024. The reported parameters were mean age at time of surgery, gravidity at time of surgery and at time of retrospective analysis, postoperative hospital stay, perioperative and postoperative complications, interval to delivery, infertility treatment, pregnancy-related and obstetrical complications, mode of delivery, and status of the perineum. We compared our data to local data from Imelda Hospital and to epidemiological data from Flanders in 2022, published by the Perinatal Activity Centre [9]. Statistical analysis was performed using percentages and means.

Surgical technique 

Intravenous antibiotic prophylaxis (cefazolin 2 g and metronidazole 1.5 g; in case of penicillin allergy, clindamycin 600 mg and metronidazole 1.5 g) was administered preoperatively. A second dose of antibiotics (cefazolin 2 g or clindamycin 600 mg in case of penicillin allergy) was administered eight hours after surgery [10]. Prior to surgery, the patient was placed in a dorsal lithotomy position with the operating table in a non-tilted position. Disinfection of the operating site was performed using an iodine-containing product. The vaginal mucosa in the posterior fornix was infiltrated with a diluted ropivacaine-adrenaline solution, followed by a posterior colpotomy of 2.5 cm [11-13]. The pouch of Douglas was opened. A vNOTES port (GelPoint vPath C2A15, Applied Medical, Rancho Santa Margarita, CA, USA) was inserted transvaginally into the pouch of Douglas [8,11-13]. A pneumoperitoneum was created with a maximum intraperitoneal pressure of 10 mm Hg. Thereafter, the operating table was tilted to the Trendelenburg position. Standard endoscopic instruments, such as bipolar grasping forceps and scissors, were used through the vNOTES port, and the surgical procedure was performed. At the end, the colpotomy was sutured using Vicryl 2/0 after extraction of the specimen [11-13]. 

## Results

All women younger than 43 years undergoing fertility-sparing vNOTES were eligible for inclusion. A total of 191 patients met the inclusion criteria, eventually resulting in 42 patients with one or more pregnancies following vNOTES and a total of 52 deliveries. Table 1 provides an overview of patient characteristics, and Table 2 shows an overview of all cases. The most frequently performed procedure was vNOTES salpingectomy (n = 26, 61.9%), in all but one case for ectopic pregnancy. Other types of vNOTES were unilateral adnexectomy (n = 3, 7.1%), cystectomy (n = 7, 16.7%), salpingostomy (n = 2, 4.8%), myomectomy (n = 1, 2.4%), and one combined myomectomy and unilateral adnexectomy (n = 1, 2.4%). In addition, two diagnostic procedures were performed for pregnancy of unknown location, but no ectopic pregnancy was present. The mean age at the time of surgery was 30 years and 10 months. In our cohort, 16 (38.1%) patients were nulliparous at the time of surgery. All procedures were uncomplicated, and no postoperative complications related to vNOTES were recorded. One patient reported postoperative nausea requiring antiemetics. Another patient needed a blood transfusion due to anaemia secondary to hemoperitoneum caused by an ectopic pregnancy.

**Table 1:** Patient characteristics * Interval in months between surgery and delivery. When two or more intervals are shown for a given patient, the interval refers to the time between surgery and the second or third delivery. G, gravidity; P, pregnancy; A, miscarriage; GA, gestational age (in weeks + days); IVF, in vitro fertilisation; ICSI, intracytoplasmic sperm injection; OI IUI, ovulation induction with intra-uterine insemination; IOL, induction of labour; PPROM, preterm premature rupture of membranes; TOLAC, trial of labour after caesarean

Patient No.	Procedure	Indication	Age	Gravidity	Interval *	Conception	Mode of delivery	Start of labour	GA	Perineum	Events
1	Salpingectomy, left	Ectopic pregnancy	28y, 7m	G4P3A1	13 months	Spontaneous	Vaginal	IOL	40+2	Episiotomy	-
					42 months	Spontaneous	Vaginal	Spontaneous	39+5	Episiotomy	
2	Salpingectomy, right	Ectopic pregnancy	27y, 3m	G4P2A2	14 months	ICSI	Primary caesarean, repeat	-	38+5	-	-
3	Salpingectomy, right	Ectopic pregnancy	32y, 5m	G4P3A1	10 months	Spontaneous	Vaginal	Spontaneous	40+0	Grade 1 rupture	-
					23 months	Spontaneous	Vaginal	IOL	40+4	Intact	-
4	Salpingectomy, right	Ectopic pregnancy	29y, 7m	G3P2A1	14 months	Spontaneous	Primary caesarean, repeat	-	39+0	-	-
5	Salpingectomy, left	Ectopic pregnancy	26y, 9m	G2P1A1	10 months	Spontaneous	Vaginal	Spontaneous	39+4	Episiotomy	-
6	Salpingectomy, left	Ectopic pregnancy	22y, 7m	G4P2A2	22 months	Spontaneous	Vaginal	Spontaneous	40+2	Episiotomy	Vacuum
					70 months	Spontaneous	Vaginal	IOL	38+3	Intact	Vacuum
7	Salpingectomy, right	Ectopic pregnancy	34y, 8m	G3P2A1	24 months	IVF	Vaginal	IOL	38+3	Grade 1 rupture	-
8	Salpingectomy, left	Ectopic pregnancy	24y, 10m	G4P2A2	26 months	Spontaneous	Vaginal	Spontaneous	37+2	Episiotomy	Tocolysis + corticosteroids
					74 months	Spontaneous	Primary caesarean, breech	-	39+6	-	
9	Salpingectomy, left	Ectopic pregnancy	34y, 7m	G3P2A1	13 months	Spontaneous	Vaginal	IOL	38+0	Episiotomy	Vacuum
					37 months	Spontaneous	Vaginal	IOL	41+3	Episiotomy	Postpartum haemorrhage
10	Diagnostic	Pregnancy of unknown location	29y, 0m	G2P1A1	18 months	IVF	Vaginal	Spontaneous	40+4	Episiotomy	-
11	Myomectomy + adnexectomy, right	Myoma + dermoid cyst	34y, 3m	G2P2A0	37 months	Spontaneous	Vaginal	Spontaneous	39+1	Episiotomy	TOLAC
12	Adnexectomy, left	Cystadenoma	37y, 6m	G3P2A1	11 months	Spontaneous	Vaginal	IOL	37+2	Grade 1 rupture	-
					37 months	Spontaneous	Vaginal	Spontaneous	38+0	Intact	
13	Salpingectomy, right	Ectopic pregnancy	23y, 4m	G5P3A2	12 months	Spontaneous	Vaginal	Spontaneous	38+5	Grade 1 rupture	
					40 months	Spontaneous	Vaginal	Spontaneous	39+0	Intact	
					77 months	Spontaneous	Vaginal	Spontaneous	37+5	Intact	
14	Salpingectomy, right	Ectopic pregnancy	26y, 5m	G4P2A2	11 months	IVF	Secondary caesarean, CPD	IOL	41+1	-	
					52 months	IVF	Primary caesarean, repeat	-	37+0	-	
15	Myomectomy	Myoma	39y, 9m	G2P1A1	81 months	IVF	Secondary caesarean, foetal distress	Spontaneous	37+0	-	PPROM, tocolysis + corticosteroids
16	Salpingectomy, left	Ectopic pregnancy	28y, 0m	G3P1A2	11 months	Spontaneous	vaginal	IOL	38+2	Episiotomy	Vacuum, vaginal hematoma
17	Salpingostomy, left	Ectopic pregnancy	28y, 7m	G3P2A1	11 months	Spontaneous	Secondary caesarean, CPD	IOL	40+0	-	TOLAC
18	Salpingectomy, left	Ectopic pregnancy	24y, 7m	G2P1A1	79 months	Spontaneous	Primary caesarean, acute fatty liver	-	35+4	-	-
19	Adnexectomy, right	Dermoid cyst	34y, 9m	G2P1A1	17 months	Spontaneous	Secondary caesarean, foetal distress	IOL	38+5	-	-
20	Salpingectomy, right	Ectopic pregnancy	29y, 4m	G5P2A3	16 months	Spontaneous	Vaginal	Spontaneous	39+4	Episiotomy	-
21	Cystectomy, left	Cystadenoma	38y, 11m	G4P3A1	13 months	Spontaneous	Vaginal	IOL	40+4	Grade 1 rupture	-
22	Salpingectomy, right	Ectopic pregnancy	31y, 0m	G5P3A2	19 months	Spontaneous	Vaginal	Spontaneous	40+3	Intact	-
23	Salpingectomy, right	Ectopic pregnancy	30y, 0m	G3P2A1	12 months	OI IUI	Vaginal	Spontaneous	37+6	Episiotomy	-
24	Salpingectomy, left	Ectopic pregnancy	31y, 10m	G4P2A2	14 months	Spontaneous	Vaginal	IOL	40+3	Episiotomy	-
25	Cystectomy, left	Paratubal cyst	29y, 10m	G1P0A0	55 months	Spontaneous	Vaginal	IOL	39+0	Intact	-
26	Salpingectomy, left	Ectopic pregnancy	28y, 6m	G4P2A2	10 months	Spontaneous	Vaginal	Spontaneous	40+0	Grade 1 rupture	-
27	Salpingectomy, right	Ectopic pregnancy	33y, 1m	G3P2A1	10 months	OI IUI	Primary caesarean, breech	-	37+6	-	-
28	Salpingectomy, left	Ectopic pregnancy	35y, 2m	G3P2A1	13 months	ICSI	Vaginal	Spontaneous	38+5	Episiotomy	TOLAC
29	Cystectomy, right	Dermoid cyst	27y, 8m	G2P2A0	17 months	Spontaneous	Vaginal	IOL	40+4	Grade 1 rupture	-
30	Salpingectomy, left	Ectopic pregnancy	26y, 7m	G3P1A2	29 months	ICSI	Primary caesarean, breech	-	39+2	-	-
31	Salpingectomy, left	Ectopic pregnancy	34y, 9m	G5P3A2	6 months	Spontaneous	Primary caesarean, repeat	-	38+1	-	-
32	Cystectomy, right	Dermoid cyst	31y, 8m	G3P1A2	23 months	OI IUI	Vaginal	Spontaneous	40+1	Grade 1 rupture	-
33	Salpingectomy, right	Ectopic pregnancy	32y, 7m	G4P3A1	10 months	Spontaneous	Primary caesarean, repeat	-	34+5	-	-
					37 months	Spontaneous	Primary caesarean, repeat	-	38+2	-	
34	Diagnostic	Pregnancy of unknown location	35y, 3m	G4P2A2	13 months	Spontaneous	Secondary caesarean, foetal distress	Spontaneous	40+1	-	TOLAC
35	Adnexectomy, right	Dermoid cyst	33y, 2m	G4P2A2	11 months	Spontaneous	Vaginal	Spontaneous	40+6	Intact	-
36	Cystectomy, right	Dermoid cyst	31y, 11m	G3P2A1	20 months	Spontaneous	Vaginal	IOL	39+4	Grade 1 rupture	-
37	Salpingectomy, left	Hydrosalpinx	33y, 1m	G2P2A0	15 months	Spontaneous	Vaginal	Spontaneous	40+4	Intact	-
38	Salpingectomy, left	Ectopic pregnancy	38y, 3m	G3P2A1	23 months	IVF	Secondary caesarean, foetal distress	IOL	40+2	-	-
39	Cystectomy, right	Dermoid cyst	23y, 10m	G2P1A1	14 months	Spontaneous	Vaginal	IOL	40+0	Episiotomy	Vacuum
40	Salpingostomy, left	Ectopic pregnancy	32y, 5m	G3P2A1	10 months	Spontaneous	Vaginal	IOL	39+1	Grade 1 rupture	Tocolysis + corticosteroids
41	Cystectomy, right	Dermoid cyst	30y, 2m	G2P2A0	13 months	Spontaneous	Primary caesarean, repeat	-	39+1	-	-
42	Salpingectomy, right	Ectopic pregnancy	30y, 3m	G3P2A1	11 months	Spontaneous	Vaginal	Spontaneous	39+2	Intact	-

**Table 2:** Overview cases vNOTES, vaginal natural orifice transluminal endoscopic surgery

Parameter	Value
Age mean, years and months	30y 10m
Nulliparous at time of surgery, n (%)	16 (38.1)
Indication for vNOTES	
Adnexectomy, n (%)	3 (7.1)
Cystectomy, n (%)	7 (16.7)
Diagnostic, n (%)	2 (4.8)
Salpingectomy, n (%)	26 (61.9)
Salpingostomy, n (%)	2 (4.8)
Myomectomy, n (%)	1 (2.4)
Myomectomy + adnexectomy, n (%)	1 (2.4)
Duration of hospitalisation	
< 24h, n (%)	18 (42.9)
≥ 24h, n (%)	26 (61.9)
Mean interval to delivery, months	19m
Fertility treatment	
Total, n (%)	12 (23.1)
Mode of delivery	
Spontaneous labour, n (%)	22 (42.3)
Vaginal delivery, n	20 (90.9)
Vacuum, n	1 (5.0)
Secondary caesarean section, n	2 (9.1)
Induction of labour, n (%)	19 (36.5)
Vaginal delivery, n	15 (78.9)
Vacuum, n	4 (21.1)
Emergency caesarean section, n	4 (21.1)
Trial of labour after caesarean, n (%)	4
Elective caesarean section, n (%)	11 (21.2)
Emergency caesarean section, n (%)	6 (11.5)
Perineum	
Intact, n (%)	10 (28.6)
Mediolateral episiotomy, n (%)	15 (42.8)
Grade 1 or 2 rupture, n (%)	10 (28.6)

Among 42 patients, 76.2% became pregnant within the first year, and 88.1% within the first 18 months after vNOTES. Eleven (26.2%) patients required assisted reproductive technology (ART), resulting in 12 pregnancies. More specifically, ovulation induction combined with timed intercourse (n = 1), ovulation induction combined with intrauterine insemination (n = 2), in vitro fertilisation (IVF) (n = 5), and intracytoplasmic sperm injection (ICSI) (n = 4). The causes for ART varied widely, with the main indications being polycystic ovary syndrome, male subfertility, and unexplained subfertility. ART had been performed to conceive prior to vNOTES surgery in 10 out of 11 patients. Among these 11 patients, eight underwent vNOTES due to an ectopic pregnancy after ART. The other procedures were performed for diagnostic purposes, myomectomy, or cystectomy due to a mature cystic teratoma.

The pregnancy-related complications varied widely and were hyperemesis gravidarum (n = 2), gestational diabetes mellitus (n = 6), of which two required insulin therapy; hypothyroidism (n = 7); gestational hypertension/pre-eclampsia (n = 6); preterm contractions (n = 3); intrahepatic cholestasis of pregnancy (n = 1); acute fatty liver (n = 1); and preterm premature rupture of membranes (PPROM) (n = 1). In the group with preterm contractions, tocolysis and foetal lung maturation with corticosteroids were administered. None of these three patients delivered preterm.

Full-term (gestational age ≥ 37 weeks) deliveries occurred in 94.2% of patients. In total, three patients delivered preterm due to acute fatty liver or PPROM, followed by partial placental abruption, and one patient had a termination of pregnancy due to cerebral and cerebellar malformation. 

Vaginal delivery was performed in 35 (67.3%) cases, of which five were vacuum-assisted, and caesarean section (CS) in 17 (32.7%) cases, subdivided into elective CS (n = 11, 21.2%) and emergency CS (n = 6, 11.5%). Indications for elective CS were previous CS in seven cases, acute fatty liver in one case, and foetal malpresentation (breech position) in three cases. Emergency CS was performed due to foetal distress in four cases and cephalo-pelvic disproportion in two cases. Trial of labour after caesarean (TOLAC) was observed in four cases, resulting in two uncomplicated vaginal deliveries and two emergency CS, one due to foetal distress and the other due to cephalo-pelvic disproportion. Overall, the performed CSs appear to be attributable to underlying obstetric conditions rather than to a prior vNOTES procedure.

In 19 cases, labour was induced, mainly as a result of hypertension/pre-eclampsia or prolonged pregnancy. The other cases were either spontaneous labour (n = 22) or planned elective CS (n = 11).

The vulvo-perineal region was evaluated after all 35 vaginal deliveries. The perineum was intact in 10 (28.6%) cases, a mediolateral episiotomy was performed in 15 (42.8%) cases, and a perineal rupture was seen in 10 (28.6%) cases. All ruptures involved grade 1 or 2 perineal tears; no grade 3 or 4 ruptures were noted. No complications related to the posterior colpotomy were observed. One vaginal delivery was complicated by a vaginal hematoma after a mediolateral episiotomy, unrelated to the posterior fornix. A revision under general anaesthesia was performed. Another case was complicated by postpartum haemorrhage due to placental retention and uterine atony.

## Discussion

In recent years, vNOTES is an emerging minimally invasive surgical technique that is used for a broad range of gynaecological indications. The rapid development of this technique is mainly due to its benefits compared with conventional laparoscopy. The safety and feasibility of vNOTES have been extensively studied and established [3-6]. Reduced operation time, improved postoperative patient comfort, and reduced hospitalisation time have been described as the main advantages [1-3]. Furthermore, the use of a posterior colpotomy leads to scar-free surgery and eliminates the risk of trocar-related complications on the abdominal wall [1-3].

The literature regarding pregnancy outcomes after vNOTES fertility-sparing surgery is limited. Chen and Ding published a case report regarding a vNOTES ovarian cystectomy for a mature ovarian teratoma in a second-trimester pregnant woman [14]. No peroperative or postoperative complications were reported, and vaginal delivery was uncomplicated at term. Our systematic search resulted in two other case series, in which no adverse outcomes were reported in five and seven patients, respectively, delivering vaginally after previous vNOTES [15-16]. Rovio and Heinonen studied 14 cases, of which 11 patients delivered vaginally, and reported no events during pregnancy or delivery [17]. They suggest a similar pregnancy rate after vNOTES compared with abdominal or laparoscopic myomectomy. Another study evaluated 17 pregnancies after vNOTES for cholecystectomy or gastrointestinal tract surgery and observed no negative impact related to the posterior colpotomy [18].

Data regarding pregnancy and vaginal delivery after other types of vaginal surgery are sparse. In general, recommendations about vaginal delivery after prolapse surgery are lacking, but often a CS is performed. Boyd et al. [19] described 11 vaginal deliveries after pelvic organ prolapse surgery. They included observational data after sacrospinous ligament fixation, midurethral sling, and anterior or posterior repair. Seven patients underwent posterior repair and showed no complications during pregnancy or vaginal delivery. No vaginal tearing of the scars was observed. We can assume that the risk of vaginal tearing or rupture of scars is extremely low. Also, in our study, no deep perineal injuries or scar disruption of the colpotomy scar were observed after vaginal delivery. Therefore, vaginal surgery in itself is not an indication for performing a CS.

In our study, which is a follow-up study of Tavano et al. [8], we evaluated 52 pregnancies in 42 patients, leading to data on 49 term and three preterm pregnancies and 35 vaginal deliveries. Tavano et al. described 26 pregnancies in 21 patients; these 21 patients are also included in our cohort [8]. To the best of our knowledge, this results in the largest retrospective cohort to date to assess pregnancy and obstetrical outcomes after vNOTES.

The absence of a control group, inherent to the retrospective study design, limits control of confounders and the ability to perform comparative analysis. To contextualise our findings, we compared our results with epidemiological data from Flanders, the northern region of Belgium. These data were published by the Perinatal Activity Centre in 2022 [9]. Data are shown in Table 3. 

**Table 3:** Comparison between our study cohort and epidemiological data Epidemiological data from Flanders, Belgium, published by the Perinatal Activity Centre in 2022 [9]. IVF, in vitro fertilisation; ICSI, intracytoplasmic sperm injection

Parameter	Our study cohort, n/n total (%)	Epidemiological data Flanders, n/n total (%)
Infertility treatment	12/52 (23.1)	5539/59968 (9.3)
Hormonal treatment	3/52 (5.7)	1730/59968 (2.9)
IVF/ICSI	9/52 (17.3)	3809/59968 (6.4)
Foetal breech position	3/52 (5.7)	3250/61854 (5.3)
Mode of delivery		
Vaginal	35/52 (67.3)	47874/61872 (77.4)
Vacuum assisted	5/35 (14.3)	5966/61872 (9.6)
Caesarean	17/52 (32.7)	13998/61872 (22.6)
Elective	11/52 (21.2)	7495/61872 (12.1)
Emergency	6/52 (11.5)	6503/61872 (10.5)
Start of labour		
Spontaneous	22/52 (42.3)	36 663/60914 (60.2)
Induction	19/52 (36.5)	17 106/60914 (28.1)
Elective caesarean	11/52 (21.1)	7145/60914 (11.7)
Perineum		
Intact	10/35 (28.6)	-
Mediolateral episiotomy	15/35 (42.8)	15272/47874 (31.9)
Perineal tear (grade 1 or 2)	10/35 (28.6)	-

A significantly higher percentage of women in our cohort conceived with the use of ART. This concerns 11 patients and a total of 23.1% (12/52) of all pregnancies in our cohort, compared with 9.3% in Flanders in 2022 [9]. Historically, the ectopic pregnancy rate is known to be higher following ART [20]. Our cohort consists of 42 patients, with 26 patients undergoing vNOTES for an ectopic pregnancy, which may provide an explanation for the high ART rates in our cohort. On the other hand, 8 out of 11 patients conceiving via ART underwent vNOTES surgery for an ectopic pregnancy.

Pelvic surgery can cause postoperative adhesions and may adversely affect fertility [21]. However, 10 out of 11 patients who used ART in our cohort had already used ART prior to vNOTES; thus, this cannot be fully attributed to vNOTES.

In our cohort, the following pregnancy complications were reported: hyperemesis gravidarum (3.8%), gestational diabetes mellitus (11.5%), hypothyroidism (13.5%), gestational hypertension/pre-eclampsia (11.5%), preterm contractions (5.8%), intrahepatic cholestasis of pregnancy (1.9%), acute fatty liver (1.9%), and PPROM (1.9%). Preterm delivery occurred in three cases due to indications not related to vNOTES. No complications related to previous vNOTES were reported.

Vaginal delivery occurred in 67.3% of cases, compared with 77.4% in Flanders [9]. Vacuum extraction was seen in 5 out of 35 vaginal deliveries (14.3%), which is higher than the mean rate in Flanders in 2022 (9.6%) [9]. The rate of CS was 32.7% in our cohort and was remarkably higher compared with the mean rate in Flanders in 2022 (22.6%) [9]. Our population is a high-risk group of patients with previous ectopic pregnancy and fertility-sparing surgery, most likely influencing obstetrical outcomes. The high rate of elective CS is due to breech position, acute fatty liver, or previous CS. Emergency CS was performed in 11.5% of cases, which is in line with the mean in Flanders in 2022 (10.5%) [9]. Therefore, the higher caesarean rate cannot be linked to previous vNOTES. Overall, the CS rate in Imelda Hospital (21.9%) is comparable to the CS rate in Flanders (22.6%) [9].

Despite a relatively small sample size, this remains the largest cohort to our knowledge in which pregnancy and obstetrical outcomes after vNOTES have been evaluated, supporting the generalisability of these findings. Another strength of this study is the long follow-up period of nearly 10 years, enabling assessment of obstetrical outcomes across multiple pregnancies after vNOTES surgery. Obstetrical data were available for nine patients with two or more subsequent pregnancies, including outcomes after repeated vaginal deliveries and repeated CS, providing additional insight into the long-term reproductive safety of vNOTES. Also, surgeon-related bias was minimised because all vNOTES procedures were performed by the same experienced surgeon.

This retrospective cohort study is limited by its retrospective design, which allows only descriptive statistics and does not permit adjustment for confounding factors. Another limitation is the lack of data on patients with an unfulfilled desire to conceive postoperatively. Although loss to follow-up of patients delivered in another hospital could be a concern, this is not an issue in the present study, as different centres use the same electronic medical record system. Nine out of 42 included patients (resulting in 13 pregnancies) delivered in another hospital, and full data collection was possible.

## Conclusions

We performed a single-centre retrospective cohort study to assess pregnancy and obstetrical outcomes after fertility-sparing vNOTES. No pregnancy-related complications were reported. Almost all deliveries were at term, except in a few cases in which the cause seemed unrelated to previous vNOTES. No increased rate of vaginal tears or rupture of the previous vaginal scar was found. Based on our data, we can assume that the posterior colpotomy used in fertility-sparing vNOTES is safe for full-term pregnancy and vaginal delivery. Therefore, there is no indication to perform an elective CS after vNOTES. Further research in a multicentre setting and in a larger cohort is necessary to confirm our findings.

## References

[1] Kaya C, Alay I, Cengiz H, Baghaki S, Aslan O, Ekin M, Yaşar L (2021). Conventional laparoscopy or vaginally assisted natural orifice transluminal endoscopic surgery for adnexal pathologies: a paired sample cross-sectional study. J Invest Surg.

[2] Ahn KH, Song JY, Kim SH, Lee KW, Kim T (2012). Transvaginal single-port natural orifice transluminal endoscopic surgery for benign uterine adnexal pathologies. J Minim Invasive Gynecol.

[3] Baekelandt J, De Mulder PA, Le Roy I (2021). Adnexectomy by vaginal natural orifice transluminal endoscopic surgery versus laparoscopy: results of a first randomised controlled trial (NOTABLE trial). BJOG.

[4] Baekelandt J, Kapurubandara S (2021). Benign gynaecological procedures by vaginal natural orifice transluminal endoscopic surgery (vNotes): complication data from a series of 1000 patients. Eur J Obstet Gynecol Reprod Biol.

[5] Li CB, Hua KQ (2020). Transvaginal natural orifice transluminal endoscopic surgery (vNOTES) in gynecologic surgeries: a systematic review. Asian J Surg.

[6] Chen X, Liu H, Sun D, Zhang JJ, Fan Q, Shi H, Lang J (2019). Transvaginal natural orifice transluminal endoscopic surgery for tubal pregnancy and a device innovation from our institution. J Minim Invasive Gynecol.

[7] Bulutlar E, Uluutku Bulutlar GB, Cilli LA, Kılıççı Ç, Şahin S (2025). vNOTES hysterectomy versus laparoscopic hysterectomy: experiences and outcomes in a tertiary center. Minim Invasive Ther Allied Technol.

[8] Tavano I, Housmans S, Bosteels J, Baekelandt J (2021). Pregnancy outcome after vaginal natural orifice transluminal endoscopic surgery, a first retrospective observational cohort study. Gynecol Obstet Invest.

[9] Goemaes R, Fomenko E, Laubach M (2023). Perinatal Health in Flanders - Year 2022 (Book in Dutch). Perinatale gezondheid in Vlaanderen - Jaar 2022.

[10] Chene G, Nohuz E, Mansoor A, Cerruto E, Lamblin G, Galea M, Baekelandt J (2021). Easy way to perform salpingectomy by transvaginal natural orifice transluminal endoscopic surgery (vNOTES) (with video). J Gynecol Obstet Hum Reprod.

[11] Baekelandt J, Vercammen J (2017). IMELDA transvaginal approach to ectopic pregnancy: diagnosis by transvaginal hydrolaparoscopy and treatment by transvaginal natural orifice transluminal endoscopic surgery. Fertil Steril.

[12] Baekelandt J (2018). Transvaginal natural-orifice transluminal endoscopic surgery: a new approach to myomectomy. Fertil Steril.

[13] Baekelandt J (2018). Transvaginal natural orifice transluminal endoscopic surgery: a new approach to ovarian cystectomy. Fertil Steril.

[14] Chen LY, Ding DC (2023). Vaginal natural orifice transluminal endoscopic surgery in a second-trimester pregnant woman with an ovarian teratoma. Gynecol Minim Invasive Ther.

[15] Zhang S, Dong Z, Liu J (2022). Safety and feasibility of vaginal delivery in full-term pregnancy after transvaginal-natural orifice transluminal endoscopic surgery: a case series. Front Surg.

[16] Feng D, He L (2021). Pregnancy and childbirth after transvaginal natural orifice transluminal endoscopic surgery for benign gynecological diseases. Int J Gynaecol Obstet.

[17] Rovio PH, Heinonen PK (2012). Pregnancy outcomes after transvaginal myomectomy by colpotomy. Eur J Obstet Gynecol Reprod Biol.

[18] Thomaidis P, Weltermann NJ, Seefeldt CS, Richards DC, Sauerwald A, Heiss MM, Bulian DR (2021). Transvaginal Hybrid-NOTES procedures-do they have a negative impact on pregnancy and delivery?. Langenbecks Arch Surg.

[19] Boyd B, Buono K, Novin A, Whitcomb E (2023). Pregnancy and outcomes after prolapse surgery: a case series. Urogynecology (Phila).

[20] Clayton HB, Schieve LA, Peterson HB, Jamieson DJ, Reynolds MA, Wright VC (2006). Ectopic pregnancy risk with assisted reproductive technology procedures. Obstet Gynecol.

[21] Practice Committee of the American Society for Reproductive Medicine, Society of Reproductive Surgeons (2007). Pathogenesis, consequences, and control of peritoneal adhesions in gynecologic surgery. Fertil Steril.

